# Is long-term PM_1_ exposure associated with blood lipids and dyslipidemias in a Chinese rural population?

**DOI:** 10.1016/j.envint.2020.105637

**Published:** 2020-05

**Authors:** Shuyuan Mao, Shanshan Li, Chongjian Wang, Yisi Liu, Na Li, Feifei Liu, Shuqiong Huang, Suyang Liu, Yuanan Lu, Zhenxing Mao, Wenqian Huo, Gongbo Chen, Hao Xiang, Yuming Guo

**Affiliations:** a Department of Global Health, School of Health Sciences, Wuhan University, 115# Donghu Road, Wuhan, China; b Global Health Institute, School of Health Sciences, Wuhan University, 115# Donghu Road, Wuhan, China; c Department of Epidemiology and Preventive Medicine, School of Public Health and Preventive Medicine, Monash University, Melbourne, Australia; d Department of Epidemiology and Biostatistics, School of Public Health, Zhengzhou University, Zhengzhou, Henan, China; e Department of Environmental and Occupational Health Sciences, University of Washington, Seattle, WA 98105, USA; f Hubei Provincial Center for Disease Control and Prevention, Wuhan 430079, Hubei Province, China; g Environmental Health Laboratory, Department of Public Health Sciences, University of Hawaii at Manoa, Honolulu, HI 96822, USA; h Hubei Biomass-Resource Chemistry and Environmental Biotechnology Key Laboratory, Wuhan University, 115# Donghu Road, Wuhan, China

**Keywords:** PM_1_, Dyslipidemia, Blood lipids, Rural areas, Cohort study

## Abstract

**Background:**

Air pollution has been shown to be associated with blood lipid levels. However, studies on long-term ambient particulate matter with aerodynamic diameter ≤1 μm (PM_1_) exposure in high-exposure areas are still limited. This study aimed to explore the associations among long-term PM_1_ exposure, blood lipids and dyslipidemias.

**Methods:**

Baseline data of The Henan Rural Cohort study was used in present study, including a total of 39,259 participants aged from 18 to 79 years. Daily levels of PM_1_ were estimated by a spatiotemporal model using ground-level measurements of PM_1_, satellite remote sensing data and other predictors, according to participants' home addresses. Individual exposure to PM_1_ was the 3-year average before baseline investigation. Linear regression and logistic regression models were applied to examine the associations among PM_1_, blood lipids ((total cholesterol (TC), triglyceride (TG), high-density lipoprotein cholesterol (HDL-C) and low-density lipoprotein cholesterol (LDL-C)), and prevalence of dyslipidemias.

**Results:**

The 3-year concentration of PM_1_ was 55.7 ± 2.1 μg/m^3^. Each 1 μg/m^3^ increment of PM_1_ was associated with an increase of 0.21% (95% confidence interval (CI): 0.11%–0.31%) in TC and 0.75% (95% CI: 0.61%–0.90%) in LDL-C, while decrease of 2.68% (95% CI: 2.43%–2.93%) in TG and 0.47% (95% CI: 0.35%–0.59%) in HDL-C. Each 1 μg/m^3^ increase in PM_1_ was associated with 6% (95% CI: 4%–8%), 3% (95% CI: 2%–5%) and 5% (95% CI: 3%–7%) higher risks of hypercholesterolemia, hyperbetalipoproteinemia and hypoalphalipoproteinemia. Sex, age and BMI statistically modified the associations between PM_1_ with blood lipid levels and dyslipidemias.

**Conclusions:**

Higher PM_1_ exposure was associated with adverse changes of blood lipid levels and dyslipidemias. Males, older and overweight participants were susceptive to the adverse effects of PM_1_.

## Highlights

• Rural residents in central China were exposed to high levels of PM_1_.
• PM_1_ exposure was related to increased TC and LDL-C, and decreased TG and HDL-C.
• High levels of PM_1_ was associated with higher risk of dyslipidemias.
• Males, older and overweight participants were vulnerable to adverse effects of PM_1_.

## 1 Introduction

The prevalence of dyslipidemia has been increasing and hyperbetalipoproteinemia remained one of the primary causes of risk-attributable death from 1990 to 2017 around the world (GBD, 2018). China Chronic Disease and Risk Factor Surveillance (CCDRFS) reported that the prevalence of hypercholesterolemia, hypertriglyceridemia, hypoalphalipoproteinemia and hyperbetalipoproteinemia in 2012 had significantly increased to 6.9%, 13.8%, 20.4%, and 8.1%, compared with that in 2002 (2.9%, 11.9%, 7.4% and 2.5%) (Longde, 2005, Zhang et al., 2018a). Epidemiological studies have shown abnormal lipid metabolism and blood lipids were related to total mortality, cardiovascular disease mortality, and risk of cardiovascular diseases (CVDs) (Anderson et al., 1987, Liu et al., 2001, Tirschwell et al., 2004).

Ambient particulate matter (PM) remained an increasingly crucial public health issue worldwide. Many studies indicated PM exposure can exacerbate abnormal lipid metabolism and lead to adverse health effects in China (Wang et al., 2018, Yang et al., 2018a), U.S.(McGuinn et al., 2019, Shanley et al., 2016, Yeatts et al., 2007), Iran (Poursafa et al., 2014) and Israel (Yitshak Sade et al., 2016). For instance, McGuinn et al. (2019) reported higher PM with diameter ≤ 2.5 μm (PM_2.5_) exposure corresponded to increased total cholesterol (TC), triglyceride (TG), low-density lipoprotein cholesterol (LDL-C) and high-density lipoprotein cholesterol (HDL-C). In addition, a longitudinal study in U.S. demonstrated that increased PM_2.5_ exposure accounted for higher risk for hypertriglyceridemia (Wallwork et al., 2017). PM with diameter ≤ 1.0 μm (PM_1_) is a major constituent of PM_2.5_, which accounts for over 80% of PM_2.5_ mass in China (Chen et al., 2018a, Yang et al., 2018b). PM_1_ has a bigger surface-to-mass ratio than PM_2.5_, suggesting that PM_1_ may have worse effects on human health as it contains more biological and/or chemical toxins, infiltrates deeper into lung alveoli and avoids the alveolar phagocytes(Brown et al., 2001, Delfino et al., 2011). Compared with the adverse effect of PM_2.5_ on hypertension and metabolic syndrome, similar adverse effects of PM_1_ were reported (Yang et al., 2018b, Yang et al., 2018c).

To date, most studies focused on the adverse effects of PM_2.5_ and PM_10_ on blood lipids and dyslipidemia, while few studies addressed the effects of PM_1_. A study based on an urban population reported that PM_1_ exposure was associated with higher risk of hypercholesterolemia, hypoalphalipoproteinemia and hyperbetalipoproteinemia (Yang et al., 2018a). Variation among living environment, food, household cooking energy, healthcare care services and economics contributed to different susceptibilities between urban and rural populations (Li et al., 2018, Liu et al., 2016). However, evidence of PM_1_ and blood lipid levels in rural populations are still lacking. Thereby, accurate estimation of adverse effect of PM_1_ exposure to dyslipidemia in rural population are urgently needed, especially in Chinese populations whose prevalence of dyslipidemia had reached to 40.8% (Zhang et al., 2018a).

This study was designed to explore the associations between long-term PM_1_ exposure, blood lipids and the prevalence of dyslipidemias in a rural population using the baseline data from The Henan Rural Cohort study.

## 2 Material and methods

### 2.1 Study population

**Fig. 1:**
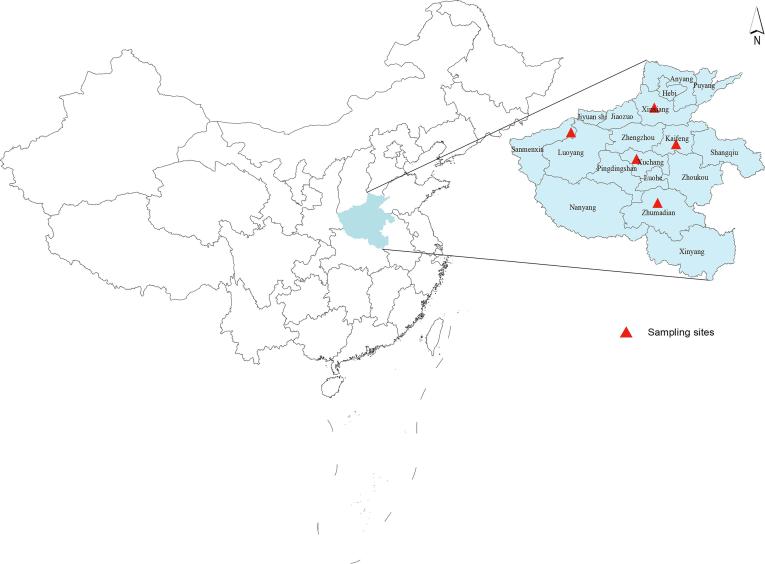
The locations of sampling sites in the Henan Rural Cohort Study.

Study population was derived from The Henan Rural Cohort study (Registration number: ChiCTR-OOC-15006699). Specific information has been reported in previous studies (Liu et al., 2019, Liu et al., 2018, Tian et al., 2018). In brief, multistage stratified random cluster sampling method was adopted to recruit the eligible participants in five rural areas (Zhumadian, Xuchang, Xinxiang, Kaifeng and Sanmenxia) in Henan province, China. The locations of sampling sites were shown in Fig. 1. A total of 39,259 permanent residents aged from 18 to 79 years were collected during 2015–2017, with a response rate of 93.7%. Individuals with severe physical or mental disease were excluded. We excluded individuals with missing lipids data (n = 138) and key demographic characteristics (n = 64). Finally, 39,057 participants were included in our analysis. Zhengzhou University Life Science Ethics Committee approved The Henan Rural Cohort study (Code: [2015] MEC (S128)). Informed consents were signed before investigation.

### 2.2 Data collection

A standardized questionnaire was designed to gather information on demographic characteristics (age, sex, marital status, incomes, education, etc.), lifestyle characteristics (smoking, alcohol drinking, diet habits, etc.) and family history (diabetes, hypertension, dyslipidemia, etc.). The survey was conducted by face-to-face interviews with well-trained workers. Moreover, a physical examination was conducted. Height (to 0.1 cm) and weight (to 0.1 kg) of each subjects were measured twice, then body mass index (BMI, kg/m^2^) was calculated. Details of the measurements and definitions have been reported in previous publications (Liu et al., 2019, Liu et al., 2018, Tian et al., 2018).

Venous blood samples were obtained from individuals after at least 8 h overnight fasting by well-trained physicians and nurses. Serum was immediately centrifuged at 3000 rpm for 10 min, 4 °C. TC, HDL-C, LDL-C and TG were measured by enzymatic methods with a chemistry analyzer (Cobas C501, Roche Diagnostics GmbH, Switzerland).

### 2.3 PM_1_ expose measurement

Concentrations of PM_1_ were estimated by a spatiotemporal model using satellite remote sensing, meteorological data and land use information at a 0.1-degree spatial resolution. Details of estimation method had been reported in previous publications (Chen et al., 2018a, Chen et al., 2018b). Briefly, we employed a general additive model to predict PM_1_ concentrations using ground-monitored PM_1_ data, two types of Moderate Resolution Imaging Spectroradiometer (MODIS) Collection 6 aerosol optical depth (AOD) data, Dark Target (DT) and Deep Blue (DB) and other spatiotemporal predictors (e.g., afforested cover, temperature, and rainfall). The adjusted coefficient of determination (R^2^) and Root Mean Squared Error (RMSE) for daily prediction was 0.58 and 21.7 µg/m^3^. Annual PM_1_ concentrations for each object were estimated according to residential addresses. Three-year average PM_1_ exposures of participants were used as an indicator of long-term PM_1_ exposure in this study, while one-year and five-year average PM_1_ exposures were used in sensitivity analyses.

### 2.4 Definition of dyslipidemias

According to the Guidelines on Prevention and Treatment of Dyslipidemia for Chinese Adults (2007), hypercholesterolemia was defined as TC ≥ 6.22 mmol/L; hypertriglyceridemia was defined as TG ≥ 2.26 mmol/L; hypoalphalipoproteinemia was defined as HDL-C < 1.04 mmol/L) and hyperbetalipoproteinemia was defined as LDL-C ≥ 4.14 mmol/L; dyslipidemia was defined as the presence of one or more abnormal blood lipid concentrations or accepting lipid-lowering medicines over the past two weeks.

### 2.5 Models and covariates

According to previous studies (McGuinn et al., 2019, Poursafa et al., 2014, Wallwork et al., 2017, Yang et al., 2018a), we include age, sex and body mass index (BMI, kg/m^2^) as covariates in baseline model. Then, we added these following factors in adjusted model, including marital status(married/cohabitation, unmarried/divorced/widowed), monthly income(<500 RMB, 500–1000 RMB, ≥1000 RMB), education level(elementary school or below, junior high school, high school or higher), smoking(never, former, current), alcohol drinking(never, former, current), family history of dyslipidemia (yes, no), high-fat diet(>75 g/day of meat from livestock and poultry) (yes, no), adequate fruit and vegetable intake(>500 g/day) (yes, no), and physical exercise(low, moderate, high).

### 2.6 Statistical analysis

Multiple linear regression analyses were performed to evaluate associations between PM_1_ exposure (per 1 μg/m^3^ increase) and naturally log-transformed blood lipids (TC, TG, LDL-C, and HDL-C), which was transformed to achieve normal distribution. And then percent differences with corresponding 95% CIs were computed by back-transforming effect estimates using 100 × [exp (β) − 1] (Yang et al., 2018a). Odds ratios (ORs) and 95% CIs were acquired from logistic regression models for dyslipidemias in respect of per 1 μg/m^3^ increment of PM_1_.

To examine potential modification effects, we performed a series of interactive analyses by including potential effect confounder, such as sex, age (<45 years, 45–60 years and ≥ 60 years), BMI (<24 kg/m^2^, 24–28 kg/m^2^ and ≥ 28 kg/m^2^) and lifestyle characteristics.

A string of sensitivity analyses were conducted to test the steadiness of our findings based on adjusted model. Associations of PM_1_ with blood lipid levels and dyslipidemias were estimated by excluding individuals who were taking lipid-lowering drugs and participants with diabetes. We also examined associations of 1-year and 5-year PM_1_ exposure with blood lipid levels and dyslipidemias.

We regarded *P* < 0.05 as statistical significance for main effects and interactions. All statistical analyses were conducted in SAS software 9.4.

## 3 Results

### 3.1 Descriptive statistics

Basic characteristics of all participants are shown in Table 1. A total of 15,365 males and 23,692 females were included in the study. The mean age of participants was 55.6 and the average BMI was 24.8 kg/m^2^. The mean level of TC, TG, HDL-C, and LDL-C was 4.75 mmol/L, 1.68 mmol/L, 1.33 mmol/L, and 2.87 mmol/L, respectively. The prevalence of hypercholesterolemia, hypertriglyceridemia, hypoalphalipoproteinemia, hyperbetalipoproteinemia and dyslipidemia in this rural population was 7.2%, 18.8%, 19.1%, 6.7% and 37.4%, respectively. The long-term PM_1_ exposure in our study was 55.6 ± 2.1 μg/m^3^, ranging from 48.1 μg/m^3^ to 70.9 μg/m^3^.

**Table 1:** Characteristics of participants in The Henan Rural Study.

Variables	Total (N = 39,057)	Men (n = 15,365)	Women (n = 23,692)
**Age (years), mean (SD)**	55.6 (12.2)	56.7 (12.3)	54.9 (12.1)
**BMI (kg/m^2^), mean (SD)**	24.8 (3.6)	24.5 (3.5)	25.0 (3.6)
**Marital status, n (%)**			
Married/cohabitation	35,059 (89.8)	13,819 (90.0)	21,240 (89.6)
Unmarried/divorced/widowed	3998 (10.2)	1546 (10.0)	2452 (10.4)
**Monthly income, n (%)**			
<500 Yuan	13,950 (35.7)	5571 (36.3)	8379 (35.4)
500 Yuan ~	12,849 (32.9)	4891 (31.8)	7958 (33.6)
≥1000 Yuan	12,258 (31.4)	4903 (31.9)	7355 (31.0)
**Education attainment, n (%)**			
Primary school or below	17,504 (44.8)	5202 (33.9)	12,302 (51.9)
Middle school	15,556 (39.8)	7100 (46.2)	8456 (35.7)
High school or above	5997 (15.4)	3063 (19.9)	2934 (12.4)
**Smoking status, n (%)**			
Never	28,470 (72.9)	4866 (31.7)	23,604 (99.6)
Former	3165 (8.1)	3142 (20.4)	23 (0.1)
Current	7422 (19.0)	7357 (47.9)	65 (0.3)
**Alcohol drinking status, n. (%)**			
Never	30,234 (77.4)	7219 (47.0)	23,015 (97.1)
Former	1822 (4.7)	1758 (11.4)	64 (0.3)
Current	7001 (17.9)	6388 (41.6)	613 (2.6)
**High fat diet, n (%)**	7419 (19.0)	3825 (24.9)	3594 (15.2)
**Adequate vegetable and fruit intake, n(%)**	16,292 (41.7)	6560 (42.7)	9732 (41.1)
**Physical activity, n (%)**			
Low	12,627 (32.3)	5468 (35.6)	7159 (30.2)
Moderate	14,745 (37.8)	4275 (27.8)	10,470 (44.2)
High	11,685 (29.9)	5622 (36.6)	6063 (25.6)
**Family history of hyperlipidemia, n (%)**	1379 (3.5)	447 (2.9)	932 (3.9)
**TC, mean (SD), mmol/L**	4.75 (0.98)	4.63 (0.94)	4.83 (0.99)
**TG, mean (SD), mmol/L**	1.68 (1.12)	1.66 (1.15)	1.69 (1.10)
**HDL-C, mean (SD), mmol/L**	1.33 (0.33)	1.26 (0.32)	1.37 (0.33)
**LDL-C, mean (SD), mmol/L**	2.87 (0.82)	2.83 (0.80)	2.90 (0.83)
**Dyslipidemia, n (%)**	14,602 (37.4)	6123 (39.9)	8479 (35.8)
**Hypercholesterolemia, n (%)**	2825 (7.2)	831 (5.4)	1994 (8.4)
**Hypertriglyceridemia, n (%)**	7330 (18.8)	2815 (18.3)	4515 (19.1)
**Hypoalphalipoproteinemia, n (%)**	7477 (19.1)	3910 (25.5)	3567 (15.1)
**Hyperbetalipoproteinemia, n (%)**	2624 (6.7)	890 (5.8)	1734 (7.3)

Abbreviations: SD, standard deviation; BMI, body mass index; TC, total cholesterol; TG, triglyceride; HDL-C, high-density lipoprotein cholesterol; LDL-C, low-density lipoprotein cholesterol.

### 3.2 Associations between PM_1_ and blood lipids

Table 2 summarizes associations between PM_1_ and blood lipids. In baseline and adjusted models, higher PM_1_ exposure was associated with elevated levels of TC and LDL-C, and decreased levels of TG and HDL-C levels. In adjusted model, every 1 μg/m^3^ PM_1_ increase was associated with 0.21% (95% CI: 0.11%–0.31%) and 0.75% (95% CI: 0.61%–0.90%) higher levels of TC and LDL-C, and 2.68% (95% CI: 2.43%–2.93%) and 0.47% (95% CI: 0.35%–0.59%) lower levels of TG and HDL-C, respectively.

**Table 2:** Associations between per 1 μg/m^3^ increment of PM_1_ and blood lipid levels.

	TC	TG	HDL-C	LDL-C
	%Changes (95%CI)	%Changes (95%CI)	%Changes (95%CI)	%Changes (95%CI)
Baseline Model^a^	0.34 (0.24, 0.44)	−2.90 (−3.13, −2.66)	−0.41 (−0.52, −0.29)	0.89 (0.75, 1.03)
Adjusted Model^b^	0.21 (0.11, 0.31)	−2.68 (−2.93, −2.43)	−0.47 (−0.59, −0.35)	0.75 (0.61, 0.90)

Abbreviations: CI, confidence interval; TC, total cholesterol; TG, triglyceride; HDL-C, high-density lipoprotein cholesterol; LDL-C, low-density lipoprotein cholesterol.

a Covariates included age, sex and BMI.

b Covariates included age, sex, BMI, education, marital status, family income, smoking, alcohol drinking, high fat diet, adequate vegetable and fruit intake, physical activities and family history of dyslipidemia.

**Fig. 2:**
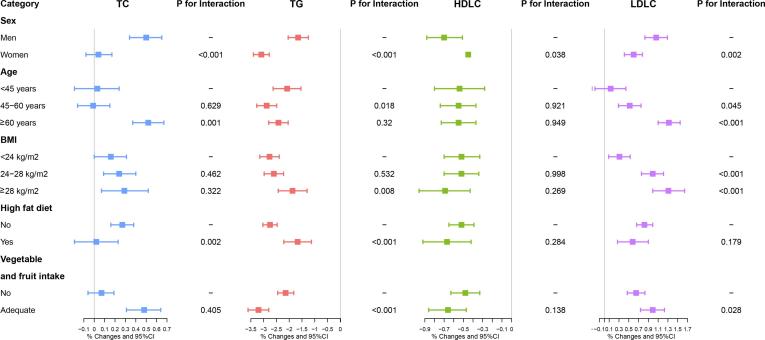
Interactions of sex, age, BMI and lifestyle characteristics on associations between a 1 μg/m^3^ increment of PM_1_ and blood lipid levels. Abbreviations: CI, confidence interval; TC, total cholesterol; TG, triglyceride; HDL-C, high-density lipoprotein cholesterol; LDL-C, low-density lipoprotein cholesterol. Covariates included age, sex, BMI, education, marital status, family income, smoking, alcohol drinking, high fat diet, adequate vegetable and fruit intake, physical activities and family history of dyslipidemia.

We have examined interactions of sex, age, BMI and lifestyle characteristics **(**see Fig. 2 and Table S1 in the Supplements**)**. The estimated effects of PM_1_ were stronger in males than females (*P* < 0.05). The interactions of age were presented in associations between PM_1_ with TC, TG and LDL-C. The association between PM_1_ and TC among participants over 60 years old (0.52%) was statistically higher than those<45 years old (0.03%). Higher effect of PM_1_ on TG was observed among participants aged from 45 to 60 compared to younger participants. Furthermore, the adverse effects of PM_1_ on LDL-C were getting worse along with the increasing of age and BMI. The modification of BMI on the effects of PM_1_ was presented on LDL-C only. Our results also showed that high-fat diet can mitigate the associations of PM_1_ with TC and TG. Adequate Vegetable and fruit intake modified the associations of PM_1_ with TG and LDL-C.

### 3.3 Associations between PM_1_ and dyslipidemias

**Fig. 3:**
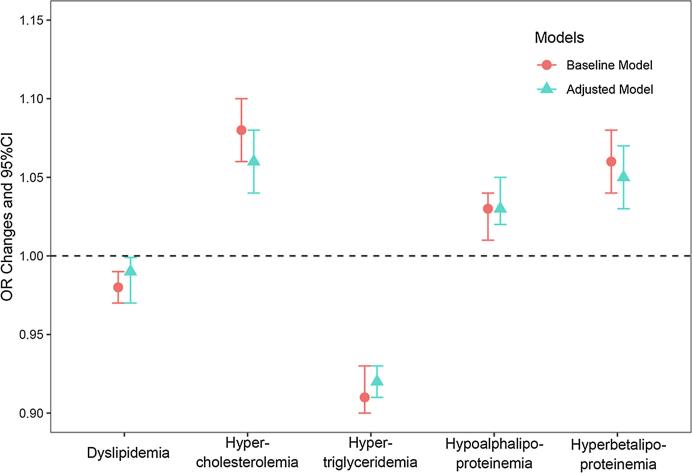
Associations between per 1 μg/m^3^ increment of PM_1_ and dyslipidemias. Abbreviations: OR, odds ratio; CI, confidence interval. Baseline Model: Covariates included age, sex and BMI; Adjusted Model: Covariates included age, sex, BMI, education, marital status, family income, smoking, alcohol drinking, high fat diet, adequate vegetable and fruit intake, physical activities and family history of dyslipidemia.

Fig. 3 presents the associations between PM_1_ and dyslipidemias. In baseline and adjusted models, increased PM_1_ exposure was associated with increased risk of hypercholesterolemia, hyperbetalipoproteinemia and hypoalphalipoproteinemia, and reduced risk of dyslipidemia and hypertriglyceridemia (see Fig. 3 and Table S2 in the Supplements). In adjusted model, the odds ratio of dyslipidemia, hypercholesterolemia, hyperbetalipoproteinemia, hypoalphalipoproteinemia, and hypertriglyceridemia was 0.99 (95% CI: 0.97–0.99), 1.06 (95% CI: 1.04–1.08), 1.03 (95% CI: 1.02–1.05), 1.05 (95% CI: 1.03–1.07), and 0.92 (95% CI: 0.91–0.93), respectively.

Table 3 displays the interaction analyses between PM_1_ and dyslipidemias of sex, age, BMI and lifestyle characteristics. Similar to the associations between PM_1_ and blood lipid levels. Males were more susceptible to PM_1_ exposure than females. For example, compared with the effect of PM_1_ on hypercholesterolemia in females (OR = 1.04, 95% CI: 1.02–1.07), higher effect in males (OR = 1.11, 95% CI: 1.08–1.15) was observed. The effects of PM_1_ on dyslipidemia and hyperbetalipoproteinemia were significantly modified by age. The effect of PM_1_ on hyperbetalipoproteinemia among participants aged over 60 years old (OR = 1.08, 95% CI: 1.04–1.12) was higher than the effect on younger participants (≤45 years old) (OR = 1.01, 95% CI: 0.95–1.07). Additionally, associations between PM_1_ and hypertriglyceridemia among overweight and obese participants were slightly weaker than the other participants. Vegetable and fruit intake modified the associations of PM_1_ with hypercholesterolemia and hypertriglyceridemia.

**Table 3:** Interactions of sex, age and BMI on associations between a 1 μg/m^3^ increment of PM_1_ and dyslipidemias.

	Dyslipidemia	P_interaction_	Hypercholesterolemia	P_interaction_	Hypertriglyceridemia	P_interaction_	Hypoalphalipo-proteinemia	P_interaction_	Hyperbetalipo-proteinemia	P_interaction_
	OR (95%CI)		OR (95%CI)		OR (95%CI)		OR (95%CI)		OR (95%CI)	
**Sex**										
Men	1.03 (1.02, 1.05)		1.11 (1.08, 1.15)		0.96 (0.94, 0.98)		1.05 (1.03, 1.07)		1.10 (1.06, 1.13)	
Women	0.96 (0.95, 0.97)	＜0.001	1.04 (1.02, 1.07)	0.001	0.91 (0.89, 0.92)	＜0.001	1.03 (1.01, 1.05)	0.097	1.03 (0.99, 1.05)	＜0.001
**Age**										
＜45 years	1.00 (0.98, 1.03)		1.05 (0.99, 1.11)		0.94 (0.91, 0.97)		1.04 (1.01, 1.07)		1.01 (0.95, 1.07)	
45–60 years	0.97 (0.96, 0.99)	0.035	1.03 (1.00, 1.07)	0.618	0.92 (0.90, 0.94)	0.300	1.04 (1.02, 1.06)	0.859	1.02 (0.98, 1.05)	0.808
≥60 years	1.00 (0.99, 1.02)	0.931	1.09 (1.06, 1.12)	0.294	0.93 (0.91, 0.95)	0.470	1.04 (1.02, 1.06)	0.847	1.08 (1.05, 1.11)	0.036
**BMI**										
＜24 kg/m^2^	0.99 (0.97, 1.01)		1.08 (1.05, 1.12)		0.90 (0.88, 0.93)		1.03 (1.00, 1.05)		1.04 (1.01, 1.08)	
24–28 kg/m^2^	0.99 (0.97, 1.00)	0.733	1.06 (1.03, 1.09)	0.406	0.93 (0.91, 0.95)	0.116	1.04 (1.02, 1.06)	0.558	1.04 (1.01, 1.07)	0.897
≥28 kg/m^2^	0.99 (0.97, 1.02)	0.942	1.04 (1.00, 1.09)	0.179	0.95 (0.93, 0.97)	0.006	1.05 (1.03, 1.08)	0.147	1.08 (1.03, 1.12)	0.231
**High fat diet**										
No	0.99 (0.97, 0.99)		1.06 (1.04, 1.08)		0.92 (0.91, 0.94)		1.04 (1.02, 1.05)		1.05 (1.03, 1.07)	
Yes	1.06 (0.98, 1.03)	0.112	1.09 (1.04, 1.14)	0.278	0.95 (0.92, 0.97)	0.096	1.04 (1.01, 1.07)	0.835	1.05 (1.00, 1.10)	0.906
**Adequate vegetable and fruit intake**										
No	1.00 (0.98, 1.01)		1.04 (1.01, 1.06)		0.95 (0.93, 0.96)		1.04 (1.02, 1.06)		1.05 (1.02, 1.08)	
Adequate	0.98 (0.96, 0.99)	0.092	1.11 (1.07, 1.15)	0.001	0.90 (0.88, 0.92)	＜0.001	1.04 (1.02, 1.06)	0.859	1.06 (1.02, 1.09)	0.692

Covariates included age, sex, BMI, education, marital status, family income, smoking, alcohol drinking, high fat diet, adequate vegetable and fruit intake, physical activities and family history of dyslipidemia.

### 3.4 Sensitivity analyses

Compared with the results of previous data analyses, associations of PM_1_ with blood lipid levels and dyslipidemias were consistent in sensitivity analyses (see Table S3 and Table S4 in the Supplements), where participants taking lipid lowering drugs and participants with diabetes were excluded, and 1-year and 5-year PM_1_ exposure were used. Table S3 and Table S4 showed the detailed results.

## 4 Discussion

This large-scale study on Chinese rural populations provides new evidence on adverse health effects of long-term PM_1_ exposure. In general, higher long-term PM_1_ exposure was associated with increased TC and LDL-C, and decreased TG and HDL-C. Higher PM_1_ exposure was associated with increased risk of hypercholesterolemia, hyperbetalipoproteinemia and hypoalphalipoproteinemia, while reduced risk of dyslipidemia and hypertriglyceridemia. Our stratified analyses suggested that males, older and overweight participants were more vulnerable to the adverse effects of PM_1_ exposure.

As PM_1_ is a major component of PM_2.5_, PM_1_ have similar adverse effects with PM_2.5_ on dyslipidemias and blood lipid levels. Compared with previous studies, the positive associations between PM, TC and LDL-C were almost accordant, but not the same. The current results were comparable but higher than Yang et al’ s findings (Yang et al., 2018a). They found every 1 μg/m^3^ increment in PM_1_ and PM_2.5_ corresponded to 0.16% (95% CI: 0.11%–0.20%) and 0.11% (95% CI: 0.08%–0.14%) increase in TC, 0.32% (95% CI: 0.26%–0.39%) and 0.29% (95% CI: 0.24%–0.35%) increase in LDL-C, and 0.14% (95% CI: 0.09%–0.18%) and 0.11% (95% CI: 0.08%–0.14%) decrease in HDL-C based on an urban population of northeast China, while our study showed that every 1 μg/m^3^ increase of PM_1_ was associated with 0.21% higher levels of TC and 0.75% of LDL-C, while 0.47% lower levels of HDL-C. This difference suggested that the rural population may be more vulnerable to PM_1_ than urban population. As far as we acknowledged, there were no other studies evaluated the associations between PM_1_ and blood lipids, and most studies focused on PM_2.5_. For example, the CATHGEN study in U.S. demonstrated that a 1 μg/m^3^ increment of PM_2.5_ was associated with an increase of 1.62%, 3.29%, and 1.70% in TC, TG, and LDL-C (McGuinn et al., 2019). Studies in the Europe (Sorensen et al., 2015) and Israel (Yitshak Sade et al., 2016) also observed that higher PM_2.5_ was associated with 0.78 mg/dL per 1 μg/m^3^ and 0.22% per 1 μg/m^3^ increased TC and LDL-C, respectively. A quantile regression analysis in U.S. suggested that participants with higher lipid levels were more vulnerable to adverse effects of PM_2.5_ (Bind et al., 2016).

However, findings on HDL-C and TG were inconsistent with previous studies. Some studies showed no associations between PM, HDL-C and risk of hypoalphalipoproteinemia (Shanley et al., 2016, Wallwork et al., 2017). However, some studies indicated that higher PM exposure was associated with lower HDL-C (Bell et al., 2017, Bind et al., 2016, Chuang et al., 2010). Wang et al. reported that 1 μg/m^3^ increment of PM_10_ was associated with 0.03% increase in HDL-C (Wang et al., 2018). In addition, our study indicated that a 1 μg/m^3^ increment in PM_1_ was associated with a 2.68% decrease levels of TG and decreased prevalence of hypertriglyceridemia (OR = 0.92, 95% CI: 0.91–0.93). However, McGuinn et al. (2019) demonstrated that 1 µg/m^3^ increment of PM_2.5_ corresponded to 3.29% increase of TG; while Yang et al. (2018a) reported no associations between PM_1_, TG and hypertriglyceridemia. Several reasons may explain these differences. First, different concentrations, sources and compositions of PM (e.g., ions, organic compound, minerals and reactive gasses) could lead to different effects (Valavanidis et al., 2008). For example, great disparities of PM pollution existed in different regions of China. The main sources of PM pollution were industrial, secondary inorganic aerosol and dust in the central of China, while that in north China were industrial, dust, and fossil fuel (Zhu et al., 2018). Second, differences in risk factor distributions among populations may account for these difference as well, such as nation, residence, lifestyle characteristics and health conditions (Cao et al., 2011). The subjects of our study were rural population. The intake of meat, dairy, eggs, fishes and shrimps intake in rural population was insufficient compared to urban population (Guo et al., 2017). In addition, the use of lipid-lowering drugs also can affect blood lipid levels. Recently a review showed that, as one of the most widely used lipid-lowering drugs in clinic, statins can significantly reduce greater levels of TC and LDL-C than TG (Awad et al., 2017). The species of lipid-lowering drugs may be a source of the associations between PM_1_ and TG while we did not collect the exact species of lipid-lowering drugs.

The mechanism of lipid metabolism and air pollution exposure remains unclear. Several potential biological pathways have been proposed from existing literature. Systemic inflammation and oxidative stress were linked to inhaled air pollution, resulting in altered lipoprotein metabolism and lipoprotein oxidation (Araujo et al., 2008, Shanley et al., 2016). Moreover, lipoprotein metabolism disorders in liver consequently emerged after inflammation of visceral adipose tissue (Poursafa et al., 2014). Experimental studies also observed that induction of systemic inflammation was linked to reduced anti-inflammatory capacity, cholesterol transport by HDL-C, and circulating adipokines (e.g. adiponectin, resistin and leptin) (Shanley et al., 2016, Xu et al., 2011a, Xu et al., 2011b, Yang et al., 2018a). Another possible mechanism is that inhaled air pollution was related to DNA methylation. A human intervention study in Shanghai provided robust human evidence that PM was associated with rapidly reduction of DNA methylation and consequently mediate its effects on cardiovascular biomarkers (Chen et al., 2016). Moreover, a cohort study in U.S. found associations between air pollution and genes methylation which were relevant to lipoprotein metabolism and systemic inflammation (Bind et al., 2014). Mendez et al.’s (Mendez et al., 2013) experimental study on mouse demonstrated that chronic inhalation exposure to PM_2.5_ caused up-regulation of genes related to lipogenesis, lipolysis, adipocyte differentiation, and lipid droplet formation (Mendez et al., 2013, Wallwork et al., 2017).

According to exist literature, various factors can modify the adverse effects of PM on blood lipid levels. Females were found to be more vulnerable to adverse effected of PM in previous studies, while the estimated effects in the current study among males were higher than females (Bell et al., 2017, Yang et al., 2018a). Vulnerabilities of biological and lifestyle characteristics between male and female might explain this difference. First, as most participants in our study were farmers. Male farmers were accustomed to have a longer outdoor working time and expose to more PM_1_ than females. Besides, Lelieveld et al. (2015) found that agriculture was an important anthropogenic source of outdoor air pollution category that contributing one-fifth premature mortality. Besides, previous literature revealed that inhaled air pollution can act as an exogenous hormones, activating estrogen-disrupting effects, and playing a crucial role in reactive oxygen species generating and oxidative stress inducting (Bell et al., 2017, Chen et al., 2013, Miller, 1994). In addition, males had a higher rate of smoking and drinking, which can cause higher risk of dyslipidemias. Overweight and obese individuals were found more susceptible to the effects of PM_1_ in our study. A multi-city analysis of PM had similar observation that the adverse effects of PM_1_ among overweight participants (BMI ≥ 25 kg/m^2^) were stronger (Yang et al., 2018a). Sun et al. (2009) reported similar results in an animal study that adipose inflammation and visceral adiposity were significantly increased in high-fat induced male mice exposed to PM_2.5_. Besides, our findings showed stronger adverse effects of PM_1_ were presented among elderly (*P* < 0.05). However, studies in U.S. and Denmark showed no modification effects of age on the association between PM and blood lipid levels (Shanley et al., 2016, Sorensen et al., 2015). Health services utilization may explain this difference. The elderly had a low level of local health services utilization in China, which may bring them in a vulnerable status to the adverse effects of air pollutants (Zhang et al., 2018b).

Dietary habits were found to be a major risk factor for cardiovascular disease and blood lipid levels. Unhealthy dietary habits increased the risk of developing dyslipidemia, such as consuming excessive amounts of sugar and fats. Our current study analyzed the interactions of dietary habits on PM_1_ with blood lipid levels and dyslipidemias. But surprisingly, high-fat diet can mitigate the associations of PM_1_ with TC and TG in our current study. The same association was reported in a Taiwanese study, Lin et al., (2019) found high-fat intake inversely correlated with high TC in unadjusted model, while the correlation became not statistically significant after adjustment for some lifestyle characteristics. Vegetable and fruit intake was found to be a modified factor as well. Studies showed that intake of fruit can alleviate the adverse health effects of air pollution, which was due to the rich content of vitamin C, carotenoids, and flavonoids in dietary fruit and vegetable, given the effects of antioxidants against inflammation and oxidative stress (Bowler and Crapo, 2002, Lin et al., 2017). Adequate vegetable and fruit intake modified the associations of PM_1_ with TG and LDL-C, but the correlations among PM_1_ and dyslipidemias were not statistically significant. Chinese people are accustomed to intake cooked vegetables, it was possible that some antioxidant components in the vegetables have been destroyed during the cooking process. Besides, our study collected insufficient information about vegetable and fruit consumption. Further studies are needed to explore the potential effects of high-fat diet and intake of fruit and vegetables for those exposed to PM_1_.

Some limitations in this study should be noted. First, a number of possible confounders were not controlled in this study, such as passive smoking, acute infection and inflammation (Nigam, 2011, Yang et al., 2018a). These information was not available in our study, which may affect the results. Second, we did not consider the effects of other air pollutants, as some air pollutants were highly correlated (Chen et al., 2017, Chen et al., 2012). Third, the concentrations of PM_1_ in our areas was comparatively concentrated, the IQR of PM_1_ was only 2.54 µg/m^3^. The sensitivity of residents in high-pollution areas often tended to be reduced as the adverse effects of PM among vulnerable subjects may have reached maximum level (Cao et al., 2011). In addition, although permanent residents who have signed informed consent were selected as participants, and those people who had moved within the 3 years were excluded before investigation, we do not acknowledge the exact move records of each individual over the past three years. Furthermore, the spatial resolution was not as accurate as models (1000 m × 1000 m and 500 m × 500 m) emerged in the last two years, which may cause measurement errors.

## 5 Conclusions

In summary, long-term exposure to high PM_1_ was associated with the changed blood lipid levels, as well as higher risk of hypercholesterolemia, hyperbetalipoproteinemia and hypoalphalipoproteinemia in Chinese rural populations. In particular, males, older and overweight participants were more vulnerable to the adverse effects of PM_1_. Our findings add new evidence on associations between PM_1_ exposure and cardiovascular diseases in rural population. Further longitudinal studies based on individual PM_1_ exposure are urgently warranted to verify our findings.

## Funding

This work was supported by the Foundation of National Key Program of Research and Development of China (Grant No: 2016YFC0900803), National Natural Science Funding of China (Grant No. 81903279), the Bill & Melinda Gates Foundation (Grant No. OOP1148464), and the Natural Science Fund of Hubei Province (Grant No: 2018CFB634). Dr. Guo is supported by Career Development Fellowship APP1107107 from the Australian National Health and Medical Research Council (NHMRC). Dr. Li is supported by Early Career Fellowship APP1109193 from the Australian NHMRC.

## CRediT authorship contribution statement

**Shuyuan Mao:** Methodology, Formal analysis, Writing - original draft, Visualization. **Shanshan Li:** Resources, Data curation, Funding acquisition. **Chongjian Wang:** Investigation, Data curation, Project administration, Funding acquisition. **Yisi Liu:** Validation, Writing - review & editing. **Na Li:** Methodology, Software. **Feifei Liu:** Methodology, Software. **Shuqiong Huang:** Validation, Supervision. **Suyang Liu:** Validation, Supervision. **Yuanan Lu:** Validation, Supervision. **Zhenxing Mao:** Investigation, Resources, Data curation. **Wenqian Huo:** Investigation, Resources, Data curation. **Gongbo Chen:** Methodology, Formal analysis, Funding acquisition. **Hao Xiang:** Conceptualization, Methodology, Writing - review & editing, Funding acquisition. **Yuming Guo:** Resources, Writing - review & editing, Funding acquisition.

## Declaration of Competing Interest

The authors declare that they have no known competing financial interests or personal relationships that could have appeared to influence the work reported in this paper.
