# Supplementary material for: Is long-term PM1 exposure associated with blood lipids and dyslipidemias in a Chinese rural population?
Source: Environ Int. 2020 May;138:105637. doi: 10.1016/j.envint.2020.105637 (PMC7152799; doi:10.1016/j.envint.2020.105637)
Supplement: Supplementary data 2 [file mmc2.docx]

**Supplemental Material**

**Is long-term PM1 exposure associated with blood lipids and dyslipidemias in a Chinese rural population?**

**Table of Contents**

**Table S1** Interactions of sex, age, BMI and lifestyle characteristics on associations between a 1μg/m^3^ increment in PM_1_ and blood lipids.

**Table S2** Interactions of lifestyle characteristics on associations between per 1μg/m^3^ increment of PM_1_ and dyslipidemias

**Table S3.** Sensitivity analysis for associations between per 1-μg/m^3^ increment of PM_1_ and blood lipids.

**Table S4.** Sensitivity Analysis for associations between per 1-μg/m^3^ increment of PM_1_ and dyslipidemias.

**Table S1 Interactions of sex, age, BMI and lifestyle characteristics on associations between a 1μg/m^3^ increment in PM_1_ and blood lipids.**

|  | **TC** | **P_interaction_** | **TG** | **P_interaction_** | **HDL-C** | **P_interaction_** | **LDL-C** | **P_interaction_** |
| --- | --- | --- | --- | --- | --- | --- | --- | --- |
|  | **%changes (95%CI)** |  | **%changes (95%CI)** |  | **%changes (95%CI)** |  | **%changes (95%CI)** |  |
| **Sex** |  |  |  |  |  |  |  |  |
| Men | 0.50 (0.34, 0.65) | - | -1.65 (-2.04, -1.25) | - | -0.70 (-0.88, -0.51) | - | 1.06 (0.83, 1.29) | - |
| Women | 0.04 (-0.08, 0.17) | ＜.001 | -3.09 (-3.40, -2.78) | ＜.001 | -0.45 (-0.60, -0.31) | 0.038 | 0.60 (0.41, 0.78) | 0.002 |
| **Age** |  |  |  |  |  |  |  |  |
| <45 years | 0.03 (-0.19, 0.24) | - | -2.08 (-2.63, -1.54) | - | -0.54 (-0.80, -0.28) | - | 0.13 (-0.19, 0.44) | - |
| 45-60 years | -0.01 (-0.16, 0.15) | 0.629 | -2.88 (-3.27, -2.49) | 0.018 | -0.55 (-0.74, -0.37) | 0.921 | 0.52 (0.29, 0.75) | 0.045 |
| ≥60 years | 0.52 (0.37, 0.67) | 0.001 | -2.42 (-2.80, -2.04) | 0.320 | -0.55 (-0.73, -0.37) | 0.949 | 1.32 (1.10, 1.55) | ＜.001 |
| **BMI** |  |  |  |  |  |  |  |  |
| <24 kg/m^2^ | 0.16 (0.00, 0.31) | - | -2.77 (-3.15, -2.39) | - | -0.52 (-0.70, -0.33) | - | 0.31 (0.09, 0.53) | - |
| 24-28 kg/m^2^ | 0.24 (0.09, 0.40) | 0.462 | -2.60 (-2.98, -2.22) | 0.532 | -0.52 (-0.70, -0.34) | 0.998 | 0.99 (0.76, 1.21) | ＜.001 |
| ≥28 kg/m^2^ | 0.29 (0.07, 0.52) | 0.322 | -1.87 (-2.43, -1.30) | 0.008 | -0.69 (-0.96, -0.43) | 0.269 | 1.31 (0.99, 1.64) | ＜.001 |
| **High fat diet** | |  |  |  |  |  |  |  |
| No | 0.27 (0.16, 0.38) | - | -2.75 (-3.03, -2.47) | - | -0.52 (-0.65, -0.39) | - | 0.82 (0.66, 0.99) | - |
| Yes | 0.02(-0.19, 0.23) | 0.002 | -1.67 (-2.21, -1.13) | ＜.001 | -0.67 (-0.92, -0.42) | 0.284 | 0.58 (0.27, 0.90) | 0.179 |
| **Vegetable and fruit intake** | | |  |  |  |  |  |  |
| No | 0.07 (-0.06, 0.19) | - | -2.13 (-2.45, -1.82) | - | -0.48 (-0.63, -0.33) | - | 0.65 (0.47, 0.83) | - |
| Adequate | 0.48 (0.31, 0.64) | 0.405 | -3.20 (-3.61, -2.80) | ＜.001 | -0.66 (-0.86, -0.47) | 0.138 | 0.99 (0.74, 1.23) | 0.028 |
| **Physical activity** | |  |  |  |  |  |  |  |
| Low | 0.56 (0.38, 0.73) | - | -2.36 (-2.79, -1.93) | - | -0.68 (-0.88, -0.48) | - | 0.80 (0.55, 1.05) | - |
| Moderate | -0.19 (-0.34, -0.04) | 0.147 | -2.56 (-2.94, -2.18) | 0.494 | -0.72 (-0.90, -0.54) | 0.768 | 0.49 (0.27, 0.71) | 0.062 |
| High | 0.43 (0.25, 0.62) | 0.124 | -2.69 (-3.15, -2.23) | 0.302 | -0.14 (-0.36, -0.08) | ＜.001 | 1.17 (0.90, 1.44) | 0.049 |
| **Smoking** |  |  |  |  |  |  |  |  |
| Never | 0.07 (-0.05, 0.18) | - | -2.99 (-3.28, -2.70) | - | -0.48 (-0.625, -0.35) | - | 0.68 (0.51, 0.84) | - |
| Former | 0.54 (0.21, 0.86) | 0.142 | -1.96 (-2.77, -1.13) | 0.018 | -0.73 (-1.12, -0.35) | 0.231 | 1.33 (0.65, 2.02) | 0.067 |
| Current | 0.68 (0.45, 0.90) | 0.663 | -0.95 (-1.51, -0.38) | ＜.001 | -0.72 (-0.98, -0.45) | 0.119 | 1.06 (0.73, 1.38) | 0.038 |
| **Drinking** |  |  |  |  |  |  |  |  |
| Never | 0.13 (0.02, 0.25) | - | -2.91 (-3.19, -2.63) | - | -0.57 (-0.70, -0.43) | - | 0.62 (0.46, 0.79) | - |
| Former | 0.74 (0.28, 1.21) | 0.791 | -1.49 (-2.66, -0.31) | 0.021 | -0.83 (-0.83, -0.37) | 0.370 | 1.31 (0.83, 1.79) | 0.007 |
| Current | 0.45 (0.23, 0.67) | 0.905 | -1.20 (-1.76, -0.63) | ＜.001 | -0.41 (-0.67, -0.15) | 0.288 | 1.12 (0.79, 1.45) | 0.007 |
| **Region** |  |  |  |  |  |  |  |  |
| Xuchang | 0.06 (-0.14, 0.27) | - | 0.17 (-0.39, 0.73) | - | -0.17 (-0.43, 0.09) | - | -0.00 (-0.33, 0.30) | - |
| Zhumadian | -1.23 (-1.68, -0.77) | ＜.001 | -4.03 (-5.21, -2.84) | ＜.001 | -0.63 (-1.20, -0.06) | 0.146 | 2.19 (1.48, 2.90) | ＜.001 |
| Kaifeng | 0.87 (0.09, 1.66) | 0.051 | -1.08 (-3.13, 1.02) | 0.257 | 1.28 (0.30, 2.27) | 0.005 | 0.27 (-0.92, 1.46) | 0.653 |
| Xinxiang | 0.15 (-0.19, 0.48) | 0.690 | -0.16 (-1.06, 0.75) | 0.547 | -0.65 (-1.07, -0.23) | 0.053 | 0.17 (-0.34, 0.69) | 0.544 |
| Sanmenxia | -2.81 (-9.87, 4.81) | 0.450 | -9.11 (-25.83, 11.83) | 0.348 | 3.20 (-6.08, 13.39) | 0.490 | -3.15 (-13.67, 8.66) | 0.588 |

Abbreviations: CI, confidence interval; TC, total cholesterol; TG, triglyceride; HDL-C, high-density lipoprotein cholesterol; LDL-C, low-density lipoprotein cholesterol.

Covariates included age, sex, BMI, education, marital status, family income, smoking, alcohol drinking, high fat diet, adequate vegetable and fruit intake, physical activities and family history of dyslipidemia.

**Table S2. Interactions of lifestyle characteristics on associations between per 1μg/m^3^ increment of PM_1_ and dyslipidemias.**

|  | **Dyslipidemia** | **P**  **_interaction_** | **Hypercholesterolemia** | **P**  **_interaction_** | **Hypertriglyceridemia** | **P**  **_interaction_** | **Hypoalphalipo-**  **proteinemia** | **P**  **_interaction_** | **Hyperbetalipo-**  **proteinemia** | **P**  **_interaction_** |
| --- | --- | --- | --- | --- | --- | --- | --- | --- | --- | --- |
|  | **OR (95%CI)** |  | **OR (95%CI)** |  | **OR (95%CI)** |  | **OR (95%CI)** |  | **OR (95%CI)** |  |
| **Physical activity** | |  |  |  |  |  |  |  |  |  |
| Low | 1.00 (0.98, 1.02) | - | 1.09 (1.06, 1.13) | - | 0.93 (0.91, 0.95) | - | 1.05 (1.03, 1.08) | - | 1.04 (1.01, 1.07) | - |
| Moderate | 0.98 (0.96, 1.00) | 0.060 | 1.00 (0.97, 1.04) | ＜.001 | 0.93 (0.91, 0.95) | 0.660 | 1.05 (1.02, 1.07) | 0.620 | 1.01 (0.98, 1.05) | 0.260 |
| High | 0.99 (0.97, 1.01) | 0.346 | 1.10 (1.06, 1.14) | 0.76 | 0.92 (0.90, 0.95) | 0.540 | 1.01 (0.98, 1.04) | 0.011 | 1.12 (1.08, 1.17) | 0.002 |
| **Smoking** |  |  |  |  |  |  |  |  |  |  |
| Never | 0.97 (0.96, 0.98) | - | 1.05 (1.02, 1.07) | - | 0.91 (0.90, 0.93) | - | 1.03 (1.02, 1.05) | - | 1.03 (1.01, 1.05) | - |
| Former | 1.01 (0.98, 1.05) | 0.025 | 1.10 (1.02, 1.17) | 0.207 | 0.91 (0.87, 0.95) | 0.947 | 1.06 (1.02,1.10) | 0.197 | 1.10 (1.03, 1.18) | 0.063 |
| Current | 1.06 (1.03, 1.08) | ＜.001 | 1.13 (1.08, 1.19) | 0.004 | 0.99 (0.96, 1.02) | ＜.001 | 1.05 (1.02, 1.08) | 0.234 | 1.12 (1.06, 1.17) | 0.002 |
| **Drinking** |  |  |  |  |  |  |  |  |  |  |
| Never | 0.98 (0.96, 0.99) | - | 1.05 (1.03, 1.07) | - | 0.91 (0.90, 0.93) | - | 1.04 (1.02, 1.05) | - | 1.04 (1.01, 1.06) | - |
| Former | 1.04 (0.99, 1.10) | 0.011 | 1.23 (1.12, 1.35) | 0.001 | 0.92 (0.86, 0.99) | 0.706 | 1.07 (1.02, 1.13) | 0.262 | 1.19 (1.09, 1.31) | 0.003 |
| Current | 1.03 (1.01, 1.06) | ＜.001 | 1.09 (1.04, 1.14) | 0.163 | 0.99 (0.96, 1.02) | ＜.001 | 1.03 (1.01, 1.06) | 0.817 | 1.09 (1.04, 1.14) | 0.064 |
| **Region** |  |  |  |  |  |  |  |  |  |  |
| Xuchang | 1.02 (0.99, 1.05) | - | 1.04 (0.96, 1.11) | - | 1.02 (0.99, 1.05) | - | 1.01 (0.98, 1.04) | - | 1.05 (0.99, 1.11) | - |
| Zhumadian | 0.90 (0.86, 0.95) | ＜.001 | 0.84 (0.75, 0.95) | 0.004 | 0.88 (0.83, 0.94) | ＜.001 | 1.01 (0.95, 1.08) | 0.987 | 1.15 (1.03, 1.29) | 0.140 |
| Kaifeng | 0.97 (0.88, 1.06) | 0.226 | 1.15 (1.01, 1.31) | 0.187 | 1.09 (0.96, 1.25) | 0.298 | 0.96 (0.84, 1.09) | 0.422 | 1.09 (0.97, 1.22) | 0.539 |
| Xinxiang | 0.99 (0.95, 1.03) | 0.157 | 0.97 (0.92, 1.03) | 0.152 | 0.98 (0.94, 1.03) | 0.236 | 1.01 (0.97, 1.06) | 0.982 | 0.99 (0.93, 1.06) | 0.217 |
| Sanmenxia | 0.66 (0.25, 1.76) | 0.378 | 0.79 (0.00, 5.84) | 0.790 | 0.74 (0.26, 2.14) | 0.554 | 1.01 (0.34, 3.06) | 0.998 | 0.86 (0.14, 5.34) | 0.829 |

Abbreviations: OR, odds ratio; CI, confidence interval.

Covariates included age, sex, BMI, education, marital status, family income, smoking, alcohol drinking, high fat diet, adequate vegetable and fruit intake, physical activities and family history of dyslipidemia.

**Table S3. Sensitivity Analysis for associations between per 1μg/m^3^ increment of PM_1_ and blood lipids.**

|  | **TC** |  | **TG** |  | **HDL-C** |  | **LDL-C** |
| --- | --- | --- | --- | --- | --- | --- | --- |
|  | **% changes (95%CI)** |  | **% changes (95%CI)** |  | **% changes (95%CI)** |  | **% Changes (95%CI)** |
| **All participants** ^a^ | 0.21 (0.11, 0.31) |  | -2.68 (-2.93, -2.43) |  | -0.47 (-0.59, -0.35) |  | 0.75 (0.61, 0.90) |
| Adjusted Region ^b^ | -0.05 (-0.21, 0.12) |  | -0.50 (-0.93, -0.06) |  | -0.27 (-0.48, -0.07) |  | 0.31 (0.06, 0.56) |
| Adjusted meteorology ^c^ | -0.25 (-0.35, -0.14) |  | -2.04 (-2.32, -1.76) |  | -0.16 (-0.29, -0.02) |  | 0.67 (0.51, 0.83) |
| **Exclude taking lipid lowering drugs** ^a^ | 0.14 (0.04, 0.25) |  | -2.68 (-2.94, -2.43) |  | -0.48 (-0.60, -0.36) |  | 0.64 (0.49, 0.79) |
| **Exclude diabetes** ^a^ | 0.15 (0.04, 0.25) |  | -2.75 (-3.01, -2.50) |  | -0.45 (-0.57, -0.32) |  | 0.65 (0.50, 0.80) |
| **Different exposure time** ^a^ | |  |  |  |  |  |  |
| 1-year average | -0.42 (-0.02, -0.01) |  | -2.29 (-2.49, -2.09) |  | -0.32 (-0.04, -0.22) |  | 0.41 (0.29, 0.53) |
| 5-year average | 0.27 (0.17, 0.37) |  | -2.60 (-2.84, -2.35) |  | -0.41 (-0.53, -0.30) |  | 0.81 (0.66, 0.95) |

Abbreviations: CI, confidence interval; TC, total cholesterol; TG, triglyceride; HDL-C, high-density lipoprotein cholesterol; LDL-C, low-density lipoprotein cholesterol.

^a^ Covariates included age, sex, BMI, education, marital status, family income, smoking, alcohol drinking, high fat diet, adequate vegetable and fruit intake, physical activities and family history of dyslipidemia.

^b^ Covariates included age, sex, BMI, education, marital status, family income, smoking, alcohol drinking, high fat diet, adequate vegetable and fruit intake, physical activities, family history of dyslipidemia and region.

^c^ Covariates included age, sex, BMI, education, marital status, family income, smoking, alcohol drinking, high fat diet, adequate vegetable and fruit intake, physical activities, family history of dyslipidemia temperature and humidity.

**Table S4. Sensitivity Analysis for associations between per 1μg/m^3^ increment of PM_1_ and dyslipidemias.**

|  | **Dyslipidemia** | **Hypercholesterolemia** | **Hypertriglyceridemia** | **Hypoalphalipo-proteinemia** | **Hyperbetalipo-proteinemia** |
| --- | --- | --- | --- | --- | --- |
|  | **OR (95%CI)** | **OR (95%CI)** | **OR (95%CI)** | **OR (95% CI)** | **OR (95%CI)** |
| **All participants** |  |  |  |  |  |
| Baseline Model ^a^ | 0.98 (0.97, 0.99) | 1.08 (1.06, 1.10) | 0.91 (0.90, 0.93) | 1.03 (1.01, 1.04) | 1.06 (1.04, 1.08) |
| Adjusted Model ^b^ | 0.99 (0.97, 0.99) | 1.06 (1.04, 1.08) | 0.92 (0.91, 0.93) | 1.03 (1.02, 1.05) | 1.05 (1.03, 1.07) |
| Adjusted Region ^c^ | 0.99 (0.98, 1.00) | 1.10 (1.08, 1.13) | 0.92 (0.90, 0.93) | 1.03 (1.02, 1.04) | 1.07 (1.05, 1.09) |
| Adjusted meteorology ^d^ | 0.98 (0.96, 0.99) | 0.99 (0.96, 1.01) | 0.94 (0.93, 0.95) | 1.02 (1.00, 1.03) | 1.05 (1.03, 1.08) |
| **Exclude taking lipid lowering drugs** ^b^ | 0.99 (0.98, 1.01) | 1.06 (1.04, 1.08) | 0.92 (0.91, 0.93) | 1.03 (1.02, 1.05) | 1.04 (1.02, 1.07) |
| **Exclude diabetes** ^b^ | 0.98 (0.97, 0.99) | 1.06 (1.04, 1.09) | 0.92 (0.90, 0.93) | 1.03 (1.02, 1.05) | 1.04 (1.02, 1.07) |
| **Different exposure time** ^b^ | |  |  |  |  |
| 1-year average | 0.97 (0.96, 0.98) | 0.98 (0.97, 1.00) | 0.93 (0.92, 0.94) | 1.03 (1.02, 1.04) | 1.02 (1.00, 1.04) |
| 5-year average | 0.99 (0.98, 0.99) | 1.07 (1.05, 1.09) | 0.93 (0.91, 0.94) | 1.03 (1.02, 1.04) | 1.05 (1.03, 1.07) |

Abbreviations: OR, odds ratio; CI, confidence interval.

^a^ Covariates included age, sex and BMI;

^b^ Covariates included age, sex, BMI, education, marital status, family income, smoking, alcohol drinking, high fat diet, adequate vegetable and fruit intake, physical activities and family history of dyslipidemia.

^c^ Covariates included age, sex, BMI, education, marital status, family income, smoking, alcohol drinking, high fat diet, adequate vegetable and fruit intake, physical activities, family history of dyslipidemia and region.

^d^ Covariates included age, sex, BMI, education, marital status, family income, smoking, alcohol drinking, high fat diet, adequate vegetable and fruit intake, physical activities, family history of dyslipidemia temperature and humidity.
